# Dysregulated circRNAs in plasma from active tuberculosis patients

**DOI:** 10.1111/jcmm.13684

**Published:** 2018-06-30

**Authors:** Zhengjun Yi, Kunshan Gao, Ruifang Li, Yurong Fu

**Affiliations:** ^1^ Department of Laboratory Medicine Key Laboratory of Clinical Laboratory Diagnostics in Universities of Shandong Weifang Medical University Weifang China; ^2^ Department of Medical Microbiology of Clinical Medicine College Weifang Medical University Weifang China

**Keywords:** biomarker, circRNA, miRNA, pathogenesis, tuberculosis

## Abstract

Endogenous circular RNAs (circRNAs) have been reported in various diseases. However, their role in active TB remains unknown. The study was aimed to determine plasma circRNA expression profile to characterize potential biomarker and improve our understanding of active TB pathogenesis. CircRNA expression profiles were screened by circRNA microarrays in active TB plasma samples. Dysregulated circRNAs were then verified by qRT‐PCR. CircRNA targets were predicted based on analysis of circRNA‐miRNA‐mRNA interaction. GO and KEGG pathway analyses were used to predict the function of circRNA. ROC curve was calculated to evaluate diagnostic value for active TB. A total of 75 circRNAs were significantly dysregulated in active TB plasma. By further validation, hsa_circRNA_103571 exhibited significant decrease in active TB patients and showed potential interaction with active TB‐related miRNAs such as miR‐29a and miR‐16. Bioinformatics analysis revealed that hsa_circRNA_103571 was primarily involved in ras signalling pathway, regulation of actin cytoskeleton, T‐ and B‐cell receptor signalling pathway. ROC curve analysis suggested that hsa_circRNA_103571 had significant value for active TB diagnosis. Circulating circRNA dysregulation may play a role in active TB pathogenesis. Hsa_circRNA_103571 may be served as a potential biomarker for active TB diagnosis, and hsa_circRNA_103571‐miRNA‐mRNA interaction may provide some novel mechanism for active TB.

## 1 INTRODUCTION

Tuberculosis (TB), caused by *Mycobacterium tuberculosis* (Mtb), remains a major public health concern.^1^ Despite advances in the effective treatment of TB in recent years, the number of annual deaths remains almost unchanged and it is estimated that TB results in the death of about 1.5 million individuals annually, making it the most prevalent bacterial infection. Early diagnosis is the key step in controlling severe disease manifestations in patients with active TB and an essential component for preventing its transmission.^2^ However, this is challenging because of the limitations of current TB diagnostic methods with respect to their specificity and sensitivity. Due to this lack of optimal methods for the detection of TB, the definition of new biomarkers that aid diagnosis early, quickly and cheaply would be of great practical value and greatly assist in reducing the burden of TB infection.^3^

Circular RNAs (circRNAs), a novel class of endogenous non‐coding RNAs (ncRNAs), play crucial roles in the regulation of gene expression by buffering their repression of mRNA targets^4^ or sequestering specific miRNAs,^5^ which have drawn increasing interest, especially with the discovery of their tissue‐specific, cell type‐specific or developmental‐stage‐specific expression.^6^ Compared with traditional linear RNA, circRNAs with covalently closed continuous loops have more resistance to RNase degradation, which allow them to be selectively enriched during sample processing and make them more suitable for using as good candidate of molecular diagnostic biomarkers than other types of RNA.^7^

Numerous publications have demonstrated that aberrant expression of circRNAs has been shown to occur in various human diseases. Studies reported the association of hsa_circ_0002062 with pulmonary hypertension,^8^ the increased expression of hsa_circRNA_104871 with rheumatoid arthritis diagnosis^9^ and HIF1α‐associated circRNA promoting the proliferation of breast cancer cells.^10^ These findings indicate that circRNAs may be significant biological molecules to understand disease mechanisms and to identify biomarkers for disease diagnosis and therapy. Recently, the involvement of circRNAs in viral infection has also been explored.^11^, ^12^ The data suggest that circRNAs may play critical role in fine‐tuning immune response against microbial infection.^13^

However, little is known about the role of circRNAs in active TB. So, microarray screening and bioinformatics were combined to investigate the differential expression profile of circulating circRNAs in active TB patients in the study. Altered changes of circRNAs may lead to the understanding of potential role of circRNAs in active TB pathogenesis and diagnosis.

## 2 MATERIALS AND METHODS

### 2.1 Study participants and preparation of plasma specimens

A total of 32 newly diagnosed active pulmonary TB patients (BC group) were consecutively recruited from Weifang No.2 People's Hospital. The diagnosis of active TB was based on compatible clinical symptoms, and at least one sputum smear or sputum culture positive. At the same time, 29 age‐ and gender‐matched healthy controls (CC group) were enrolled from the staff of Affiliated Hospital of Weifang Medical University and Weifang No.2 People's Hospital. The characteristics of all study participants were displayed in Table S1. Plasma samples from each subject were collected by centrifugation, immediately aliquoted and stored in liquid nitrogen until further use. Three samples randomly selected from each group were applied for microarray analysis, and all of the samples were used for further qRT‐PCR validation. For the study, all the TB patients had no history of radiotherapy or chemotherapy before blood collection, and patients were excluded if they had a prior history of TB, a close contact with active TB patient, or other diseases like other infectious diseases, cancer or diabetes.

All procedures were reviewed and approved by the Committee on the Ethics of Human Experiments of Weifang Medical University before the study began, and performed in strict accordance with the Declaration of Helsinki of the World Medical Association. Informed consent was obtained from all subjects.

### 2.2 RNA extraction

Total RNA was isolated from plasma with TRIzol reagent (Invitrogen, Carlsbad, CA, USA) according to the manufacturer's protocol. Total RNA concentration and quality of each sample were determined with NanoDrop ND‐1000 spectrophotometer (NanoDrop, Wilmington, DE, USA). RNA integrity and DNA contamination were measured by electrophoresis on a denaturing agarose gel. Only RNA samples with high quality were used for subsequent microarray analysis.

### 2.3 Sample labelling and microarray hybridization

Arraystar Human Circular RNA Microarray V2 (Catalog No: AS‐CR‐H‐V2.0, Arraystar Inc., MD, USA), which coverd 13 617 human circRNAs, was used to identify circRNAs with differential expression between active TB samples and controls. Briefly, total RNA from each sample was treated with RNase R (EpicentreInc., Madison, WI, USA) to remove linear RNAs and enrich circRNAs. Then, the enriched circRNAs were amplified and transcribed into fluorescently labelled cRNAs utilizing random primer according to Arraystar Super RNA Labeling protocol (Arraystar Inc., MD, USA). The labelled cRNAs were then purified with a RNeasy Mini Kit (Qiagen, Hilden, Germany). The concentration and specific activity of the labelled cRNAs (pmol Cy3/μg cRNA) were subsequently quantified with a NanoDrop ND‐1000 spectrophotometer. Only labelled sample with the yield > 1.65 μg and the specific activity >9 pmol Cy3/μg cRNA can be proceeded to the next hybridization step. Then, 1 μg of each labelled cRNA was fragmented by adding 10 × Blocking Agent (5 μL) and 25 × Fragmentation Buffer (1 μL), then the mixture was heated at 60°C for 30 minutes; finally, 2 × Hybridization buffer (25 μL) was added to dilute the labelled cRNA. The resulting hybridization solution (50 μL) was dispensed into gasket slide and then assembled to circRNA expression microarray slide. Slides were incubated for 17 hours at 65°C in an Agilent Hybridization Oven. After washing the slides, the arrays were scanned by an Agilent Scanner G2505C.

### 2.4 Microarray data analysis

Scanned array images were imported into Agilent Feature Extraction software (version 11.0.1.1) for raw data extraction. Quantile normalization of raw data and subsequent data processing were performed using R software limma package. After quantile normalization of the raw data, low intensity filtering was performed, and the circRNAs that at least 1 out of 6 samples have flags in “P” or “M” (“All Targets Value”) were kept for subsequent differential analysis. Differentially expressed circRNAs between case and controls were identified through fold change (FC) filtering and Student's *t* test. A false discovery rate‐ (FDR) adjusted *P* value was calculated. Specifically, only the circRNA that exhibited FC > 1.5 and *P* value < .05 was selected as differentially expressed ones. Scatter plot was used to assess the variation in circRNA expression, volcano plot was applied to visualize the differential circRNAs between the two groups, and hierarchical clustering was performed to show the distinguishable circRNA expression pattern among the samples.

### 2.5 qRT‐PCR

qRT‐PCR was used to confirm circRNA expression and detect potential target miRNA of circRNA. Following RNA extraction from active TB and control plasma, cDNA was synthesized with SuperScript First‐Strand Synthesis System according to the kit's instructions (Invitrogen, Carlsbad, CA,USA). Subsequently, qRT‐PCR reaction was performed on an ABI 9700 real‐time PCR System. For circRNA analysis, PCR reaction was performed in a total volume of 20 μL, including 6 μL cDNA, 10 μL 2 × SYBR Green, 0.5 μL primer forward (10 μmol/L) and 0.5 μL primer reverse (10 μmol/L) (if needed, their sequences were available). The cycling programme entailed initiation at 95°C for 10 minutes, followed by 40 cycles of 95°C for 15 seconds and 60°C for 60 seconds. β‐actin was used as internal control. For miRNA analysis, PCR reaction was performed in a total volume of 20 μL, including 2 μL cDNA, 2.5 μL 2 × SYBR Green, 1 μL primer forward (10 μmol/L) and 1 μL primer reverse (10 μmol/L) (if needed, their sequences were available). The cycling programme consisted of 95°C for 5 minutes, followed by 40 cycles of 95°C for 10 seconds, 60°C for 60 seconds and 72°C for 20 seconds. U6 was used as internal control. All PCR reactions were conducted in triplicate. Relative expression was calculated using the 2^−ΔΔCt^ method. Control reference value for each circRNA and miRNA was obtained from pooled RNA sample from 29 healthy controls.

### 2.6 Annotation and function prediction for the validated circRNAs

To further elucidate the correlations between circRNA and miRNA, we predicted circRNA/miRNA interaction using miRNA target prediction software from Arraystar, which refers to TargetScan (http://www.targetscan.org/) and miRanda (http://www.microrna.org/). The Arraystar software was used to search for miRNA response element (MREs) on the qPCR differential circRNAs, which were then used to identify putative miRNAs based on their seed sequence complementarity. MiRNA‐targeted mRNAs were predicted by TargetScan and miRanda, GO (Gene Ontology) and KEGG (Kyoto Encyclopedia of Genes and Genomes) pathway analyses were conducted to predict hsa_circRNA_103571 function based on analysis of its targeted miRNA‐mRNAs.

### 2.7 ROC curve analysis

A receiver operating characteristic curve (ROC) was plotted; the area under the curve (AUC), sensitivity and specificity were calculated to assess the value of dysregulated circRNA for active TB diagnosis.

### 2.8 Statistical analysis

The groups were compared to evaluate the statistical significance using Student's *t* test or Chi‐square test, as appropriate. Correlation between circRNAs and their predicted miRNA targets for individual donor samples were evaluated. *P* < .05 was considered statistically significant.

## 3 RESULTS

### 3.1 Identification of dysregulated circRNAs expression profile in active TB patients

**Figure 1:**
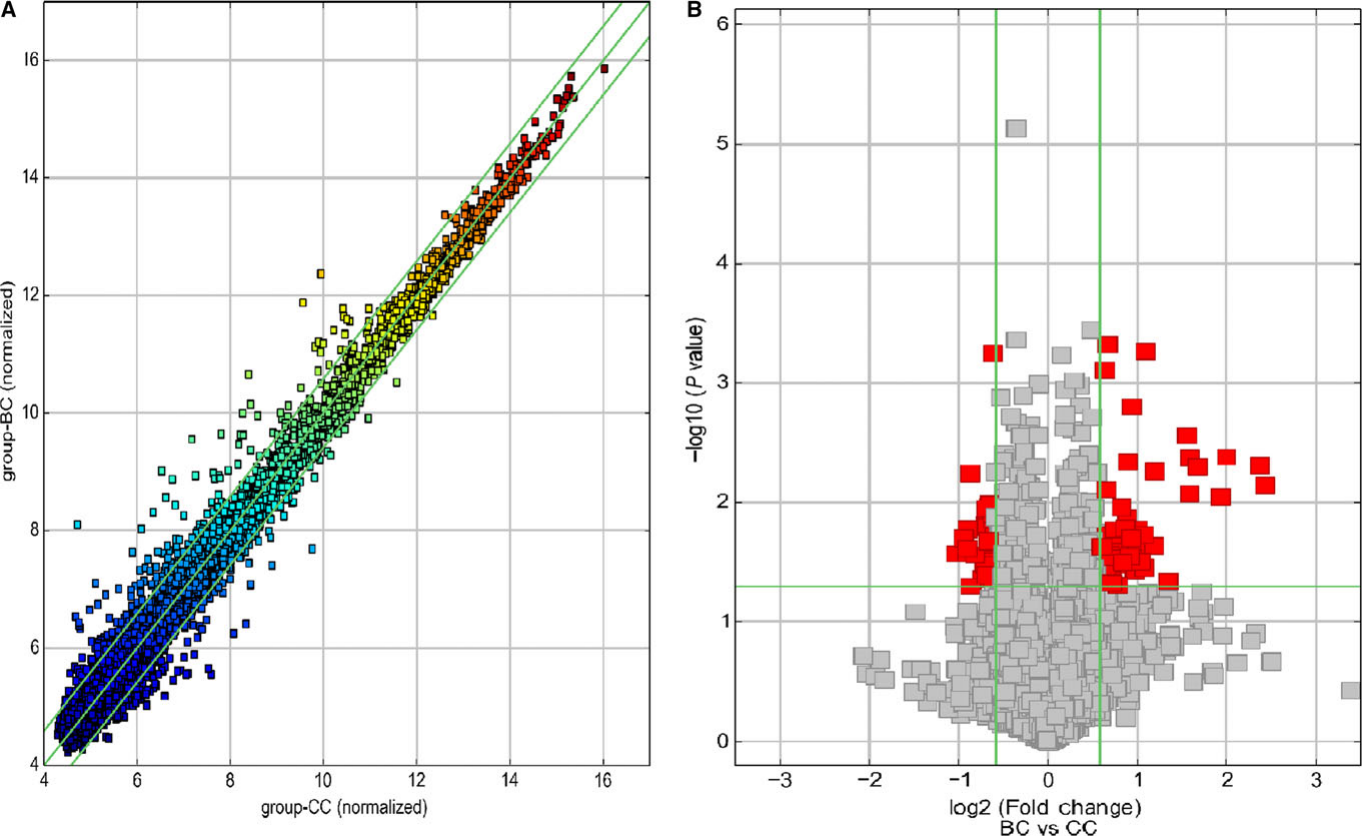
Scatter plots and volcano plots to identify differentially expressed circRNAs between active TB samples and controls. A, circRNA Scatter Plot. The values spotted in the *X* and *Y* axes represent the normalized signals of samples in the two groups. The green lines represent fold changes. The circRNAs above the upper green line and below the lower green line are those with expression fold changes >1.5 between the two groups. B, circRNA Volcano plot. The vertical lines correspond to 1.5‐fold up and down, respectively, and the horizontal line represents a *P* value of .05. The red point in the plot represents the differentially expressed circRNAs with *P* < .05. BC: active TB group; CC, control group

**Figure 2:**
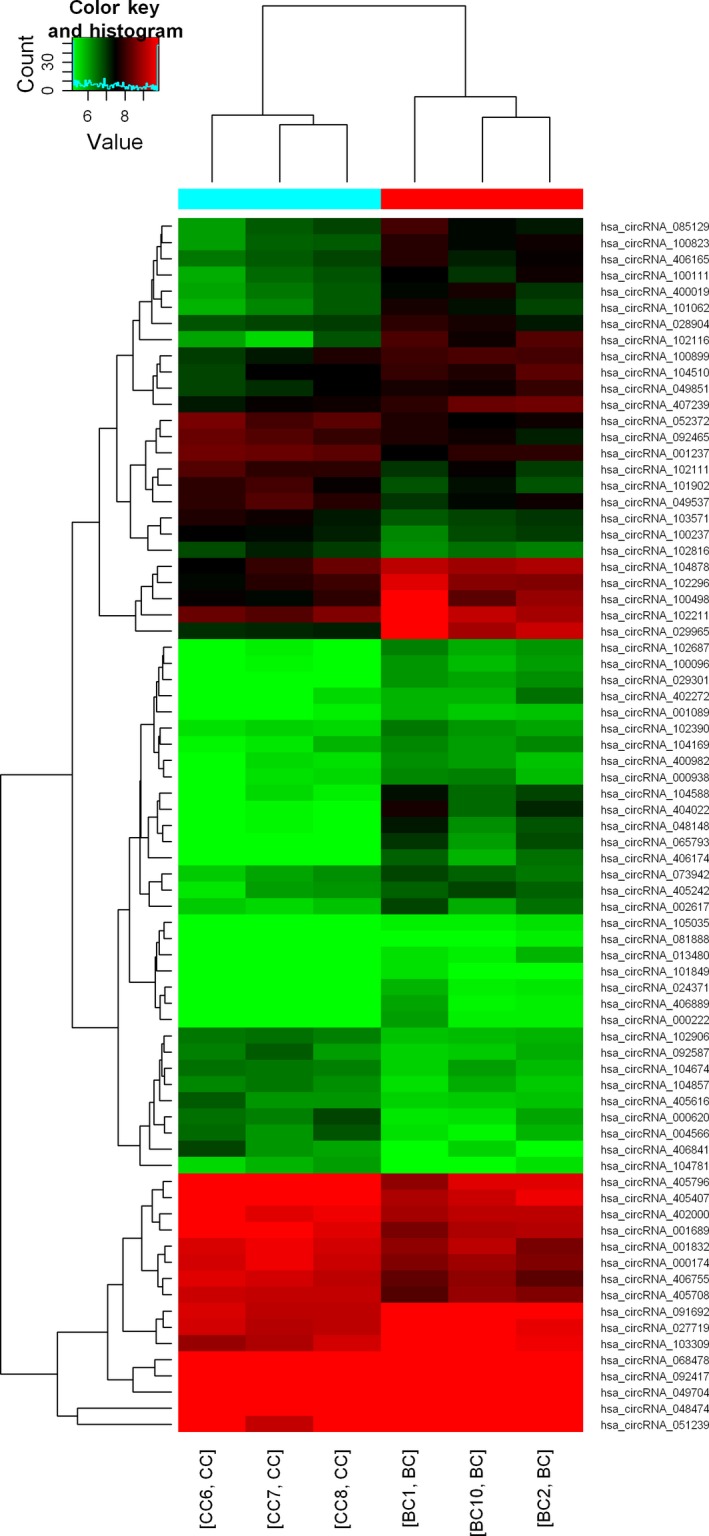
Hierarchical cluster of differentially expressed circRNAs between active TB samples and controls. The colour scale runs from green (low intensity), through black (medium intensity), to red (strong intensity). BC: active TB group (n = 3), including BC1, BC2 and BC10; CC: healthy control group (n = 3), including CC6, CC7, CC8 samples

To determine the differential circRNA expression associated with TB, three patients with active TB and three healthy controls (male/female = 2/1) were selected as the circRNA array cohort. Microarray analyses are affected by FDR as a consequence of multiple testing. Therefore, adjustment of *P* values for multiple testing has to be done. However, FDR adjusted *P* values for all circRNAs are more than .05 in the study, which means that the initial array study was underpowered to detect significant differences and more patient samples should be included for future studies of multiple circRNAs. So we presented *P* value without adjustment in the study. A total of 75 differentially expressed circRNAs were identified based on FC > 1.5 (Figure 1A) and *P* < .05 (Figure 1B), and among them, 47 circRNAs were up‐regulated and 28 were down‐regulated in active TB patients, respectively. A hierarchical cluster was created to group the dysregulated circRNAs among the samples (Figure 2). Among these circRNAs, hsa_circRNA_051239 and hsa_circRNA_406841 were the top increased and the most decreased ones, respectively. When the filter criteria were set as FC > 2 and *P* < .05, 16 circRNAs were significantly elevated and only one was obviously reduced in active TB group compared with healthy controls (Table S2). The complete microarray data in this study have been deposited in the Gene Expression Omnibus (GEO) database (the accession number GSE106953, [NCBI tracking system #18737293]).

### 3.2 Validation the differential expressed circRNAs

**Figure 3:**
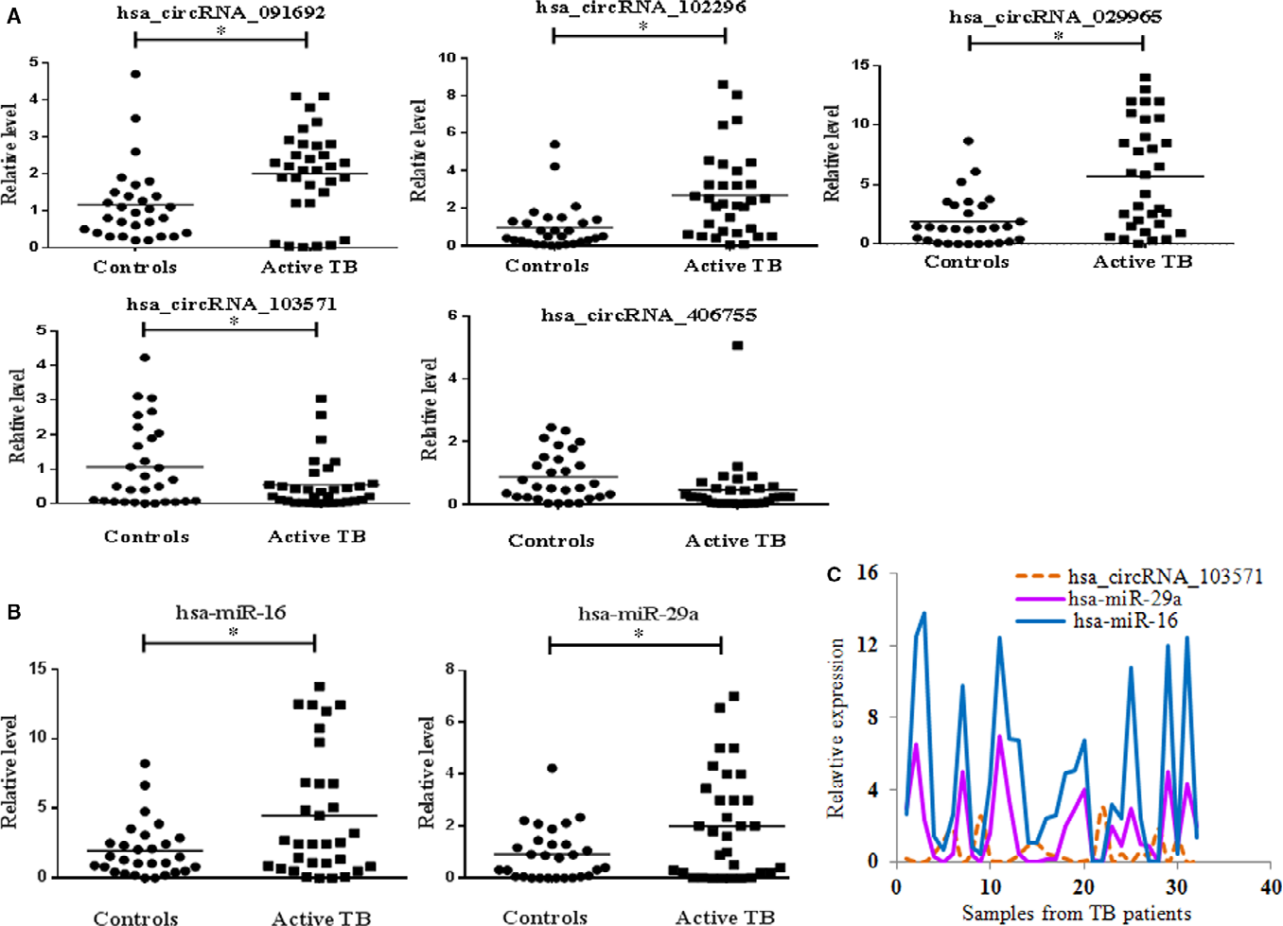
qRT‐PCR analysis of circRNAs and miRNAs A, Validation of selected dysregulated circRNAs. Expression levels of hsa_circRNA_091692, hsa_circRNA_102296 and hsa_circRNA_029965 were higher, while hsa_circRNA_103571 was lower in active TB (n = 32) compared with controls (n = 29). β‐actin was used as internal control. B, Detection of potential target miRNAs of hsa_circRNA_103571. Plasma hsa‐miR‐16 and hsa‐miR‐29a were increased in active TB (n = 32) compared with controls (n = 29). C, hsa_circRNA_103571 expression was negatively correlated with level of hsa‐miR‐29a (*r* = −.63, *P* < .05) and hsa‐miR‐16 (*r* = −.65, *P* < .05) in the patient group, respectively. U6 was used as internal control. The data were analysed using the 2^−ΔΔCt^ method and presented as relative expression levels from three independent experiments. Student's *t* test was used for statistical analysis. * *P* < .05 vs controls

To further assess the microarray expression data, we selected 6 dysregulated circRNAs, including 4 up‐regulated (091692, 102296, 029965, 100823) and 2 down‐regulated circRNAs (103571, 406755) for validation based on the following criteria: raw intensity >100, exonic‐related circRNAs, FC > 1.5 and *P* < .05. A total of 32 active TB patients and 29 healthy controls were enrolled as a validation cohort. qRT‐PCR method was used to confirm the differential expression levels from participant's plasma samples. Consistent with the microarray data, hsa_circRNA_091692, hsa_circRNA_102296, hsa_circRNA_029965 and hsa_circRNA_103571 were significantly dysregulated between cases and controls, while the expression of hsa_circRNA_406755 did not display any remarkable difference between the two groups (Figure 3A). The expression level of hsa_circRNA_100823 was not detectable in plasma sample from healthy controls.

### 3.3 Annotation and function prediction for hsa_circRNA_103571

**Figure 4:**
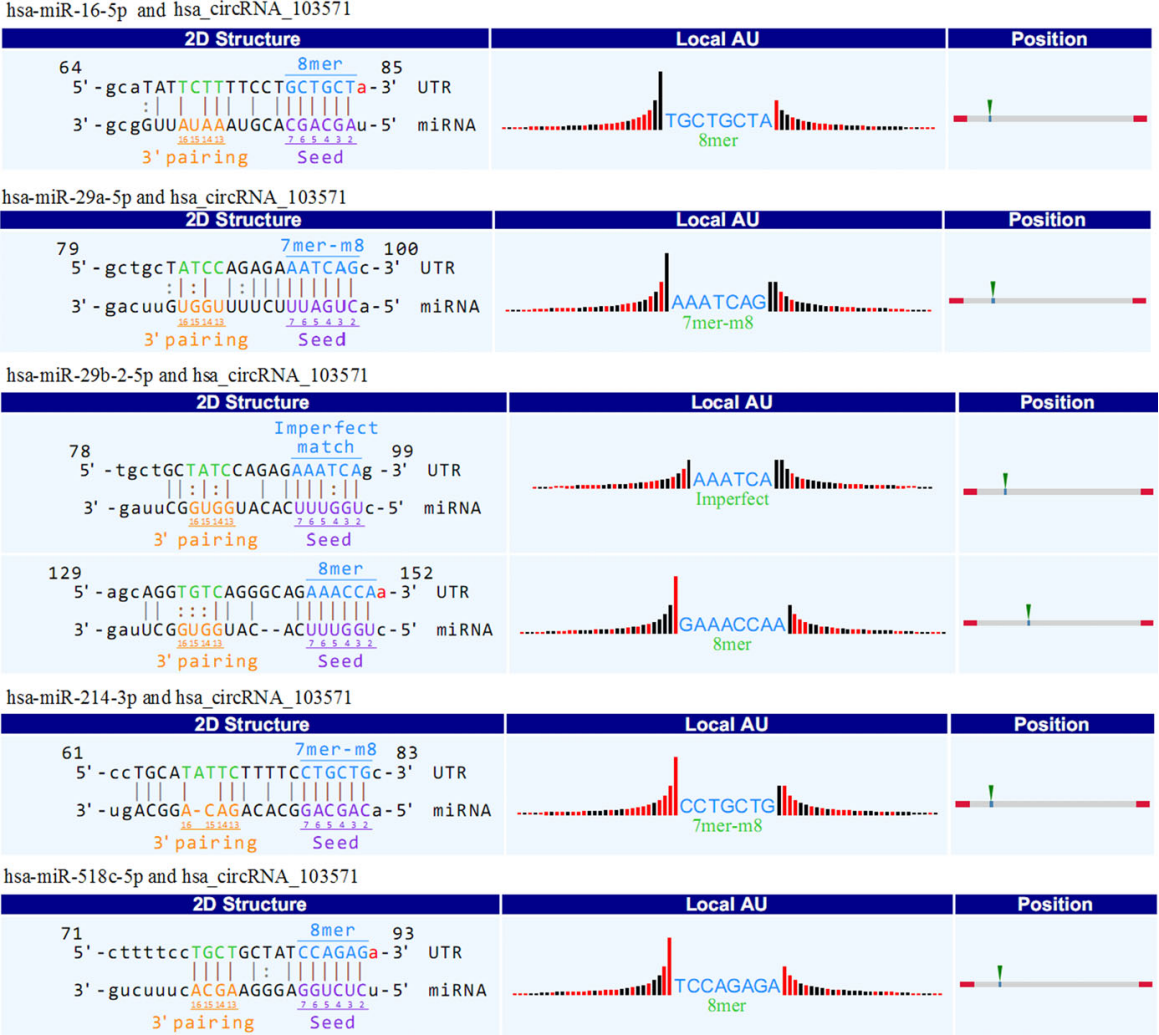
Detailed annotation of hsa_circRNA_103571 and its matched miRNAs interactions. The 2D structure displays the MRE sequence, the target miRNA seed type (7mer‐m8, 8mer, imperfect) and the pairing of target miRNA nucleotides 13‐16. The precise base positions are shown in the alignments in the upper left and right corners. The “local AU” displays the 30 nucleotides upstream and downstream of the seed sequence. Black bars stand for G/C and low accessibility. Red bars stand for A/U and high accessibility of the seed. The height of the bar represents the extent of accessibility. The position column displays the most likely relative MRE position on the linearized circRNA sequence

Recent evidences have demonstrated that some circRNAs can perform the function of “miRNA sponge” by efficiently binding and inhibiting miRNA transcription, which would further influence downstream mRNA expression and finally involved in various diseases.^14^ In the study, to investigate the potential interactions between circRNAs and its possible targeted miRNAs, we searched for MREs targeted by qRT‐PCR validated differentially expressed circRNAs. As present in Table 1, dominant MREs were targeted by the dysregulated circRNAs. Some of these miRNAs have been reported to be associated with active TB, such as miR‐29a and miR‐16, which were up‐regulated during active TB infection. It was worth noting that these miRNAs above mentioned were targeted by hsa_circRNA_103571, which was decreased in active TB group vs healthy controls in our study. We further strengthened the assumption by qRT‐PCR. As shown in Figure 3B, compared with controls, potential targets miR‐29a and miR‐16 of hsa_circRNA_103571 were increased in plasma in active TB group. Correlation analysis showed that, of 32 active TB patients, expression level of hsa_circRNA_10357` was negatively correlated with expression of miR‐29a (*r* = −.63, *P <* .05) and miR‐16 (*r* = −.65, *P <* .05), respectively (Figure 3C). In contrast, healthy controls showed no correlation between expression of hsa_circRNA_103571 and miR‐29a as well as miR‐16 (Data not shown). This indicated that hsa_circRNA_103571 might play important role in active TB pathogenesis through regulation of miRNA expression. The possible interaction information between hsa_circRNA_103571 and its matched 5 miRNAs was further annotated in detail based on sequence‐pairing prediction **(**Figure 4).

**Table 1:** The differentially expressed circRNAs and miRNA response elements

CircRNA	MRE1	MRE2	MRE3	MRE4	MRE5
hsa_circRNA_091692	hsa‐miR‐4529‐5p	hsa‐miR‐876‐3p	hsa‐miR‐4480	hsa‐miR‐6764‐3p	hsa‐miR‐4685‐5p
hsa_circRNA_102296	hsa‐miR‐578	hsa‐miR‐429	hsa‐miR‐449a	hsa‐miR‐449b‐5p	hsa‐miR‐34c‐5p
hsa_circRNA_029965	hsa‐miR‐4778‐3p	hsa‐miR‐5002‐5p	hsa‐miR‐329‐5p	hsa‐miR‐4652‐3p	hsa‐miR‐6781‐3p
hsa_circRNA_103571	hsa‐miR‐29b‐2‐5p	hsa‐miR‐29a‐5p	hsa‐miR‐518c‐5p	hsa‐miR‐214‐3p	hsa‐miR‐16‐5p

MRE, miRNA response element.

To gain further insights into the functions of hsa_circRNA_103571, we conducted the GO and KEGG analyses of its matched 5 miRNAs‐targeted mRNAs. GO analysis revealed that hsa_circRNA_103571 exhibited strong relationship with the biological process of autophagy, fatty acid biosynthetic process, regulation of actin cytoskeleton organization and so on (Table 2). Among the identified KEGG pathways, ras signalling pathway, ubiquitin‐mediated proteolysis and regulation of actin cytoskeleton were top 3, and others included wnt signalling pathway, T‐cell receptor signalling pathway, MAPK signalling pathway, B‐cell receptor signalling pathway and so on (Table S3).

**Table 2:** The significantly enriched biological processes of GO term

GO‐BP term	Gene count	*P* value
Autophagy	20	3.0E‐3
Small GTPase mediated signal transduction	47	5.7E‐3
Multicellular organism development	88	6.3E‐3
Transmembrane receptor protein tyrosine kinase signalling pathway	21	1.9E‐2
Regulation of phosphatidylinositol 3‐kinase signalling	18	1.9E‐2
Cell‐cell signalling	45	2.4E‐2
Fatty acid biosynthetic process	13	3.0E‐2
Protein glycosylation	23	3.0E‐2
Regulation of actin cytoskeleton organization	12	3.4E‐2
Vesicle fusion	14	3.5E‐2
Chemotaxis	24	3.8E‐2
Protein ubiquitination	59	4.0E‐2
Transcription, DNA‐templated	278	4.1E‐2

BP, biological processes.

### 3.4 Receiver operating characteristic analysis

**Figure 5:**
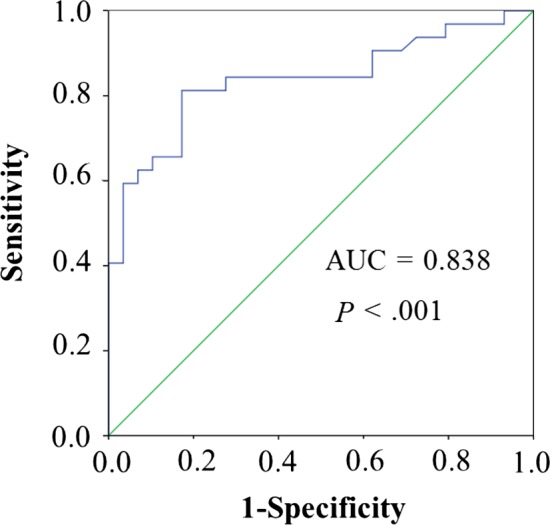
The receiver operating characteristic curve of differentially expressed hsa_circRNA_103571 in distinguishing active TB. AUC, area under the curve

To assess the potential value of confirmed hsa_circRNA_103571 for active TB diagnosis, we further performed ROC curve analysis. From this analysis, we found that ROC curve of hsa_circRNA_103571 showed a significant distinguishing efficiency with an AUC value of 0.838 (95% CI: 0.734‐0.941, *P* < .001) **(**Figure 5), which indicated that hsa_circRNA_103571 could separate patients with active TB from healthy controls.

## 4 DISCUSSION

To date, only a few studies have reported circRNAs associated with TB infection. One study has shown that circRNA expression is changed in peripheral blood mononuclear cells from active TB patients and hsa_circ_0005836 may be served as a novel potential biomarker for TB diagnosis.^15^ Another study has demonstrated that circRNAs are involved in macrophage response to Mtb infection.^16^ These findings suggest that circRNAs are worth studying for better understanding of the pathogenesis of active TB. Circulating biomarkers are more acceptable than tissue biomarkers and have greater value in clinical applications. However, until now, no publication has reported general signatures of circulating circRNAs related to active pulmonary TB. Considering the biological relevance of circRNAs to TB infection and recent studies of circRNAs in plasma, we hypothesized that cicrulating circRNAs are potentially novel biomarkers for the diagnosis and evaluation of active pulmonary TB infection.

In the study, we characterized the profile of differentially expressed circulating circRNAs in active TB patients vs healthy controls, and we found that the number of up‐regulated circRNAs was more than that of down‐regulated ones. Noticeably, when filter criteria were set as FC > 2 and *P* < .05, 17 circRNAs were identified with differential expression between samples from subjects with or without active TB, of which only one was significantly reduced in active TB group. This indicated that circulating circRNAs showed a tendency to be increased upon TB infection.

Functions of circRNAs remain largely unclear. CircRNAs may be generated from exonic or intronic sequence and function as modifiers of parental gene expression.^17^, ^18^ In the current study, we sequenced four circRNAs of interest following a microarray screening. Among them, hsa_circ_091692, hsa_circ_102296 and hsa_circ_029965 were up‐regulated in active TB plasma. Hsa_circ_091692 is encoded by the *CD99L2* (CD99‐Like 2) gene, the protein product expressed on leucocytes and play an important role in various acute and chronic inflammatory diseases.^19^ Hsa_circ_102296 aligns with *ANKRD12* (Ankyrin repeat domain 12), which encodes a 224 kD nuclear protein associated with colorectal cancer.^20^ Hsa_circ_029965 is derived from *PDS5B*, and its encoded protein is related to chromosome segregation.^21^ On the other hand, hsa_circ_103571 was down‐regulated in active TB plasma. Hsa_circ_103571 aligns with the gene *LRCH3*, which encodes a protein that may be involved in resistance to bacterial infection.^22^ However, roles of these circRNA‐related protein‐coding genes in active TB are largely unknown.

In addition to action as modifiers of parental gene expression, other intriguing possibility is that circRNAs might regulate miRNA function as miRNA sponges. Evidences are arising that circRNAs associate with related miRNAs and the circRNA‐miRNA‐mRNA axes are involved in many disease pathways. For example, it has been reported that hsa_circ_0010729 can regulate vascular endothelial cell proliferation and apoptosis via targeting miR‐186/HIF‐1α axis.^23^ So, we assumed the same mechanism for the dysregulated circRNA regulation in the development of active TB. We were interested in hsa_circ_103571, which was decreased and had good diagnostic accuracy for active TB in our study. Bioinformatics result showed that it was matched with hsa‐miR‐29a and hsa‐miR‐16, which have been reported to be up‐regulated in active TB.^24^, ^25^ Moreover, we found that hsa‐miR‐29a was also matched with hsa_circRNA_104674, which was down‐regulated in our study. Other miRNA, such as has‐miR‐33, which were matched with down‐regulated hsa_circRNA_100237 in our study, has also been reported to be increased in Mtb infection.^26^ These findings suggested that specific circRNAs, such as hsa_circ_103571, might be involved in TB pathogenesis by binding and inhibiting related miRNA transcription.

The differential expressed hsa_circ_103571 found in active TB has not been reported in the other diseases ever since, which indicates the specificity and efficiency of this circRNA as a molecular biomarker. By bioinformatics analysis of circRNA‐miRNA‐mRNA interaction, we predicted the function of hsa_circ_103571 in active TB. Results from GO process and KEGG pathway could help to enrich and identify important mRNAs. In the top dysregulated GO processes of differentially expressed mRNAs, the biological process of autophagy, fatty acid biosynthetic process and regulation of actin cytoskeleton organization were identified. As autophagy plays an important role in the development of active TB,^27^ it is possible to speculate that mRNAs might be jointly involved in this process, and the predicted mRNAs in circRNA‐miRNA‐mRNA network might be related to hsa_circ_103571 function. KEGG enrichment analysis also points to the effective signalling pathways, such as regulation of actin cytoskeleton, which has been reported to be a key mediator of mycobacterium infection.^28^

In summary, our study provided a profile of circulating circRNAs in active TB and healthy controls. The alteration of circulating circRNAs expression indicated that these dysregulated circRNAs might be involved in the onset and development of active TB. Specific circRNAs may regulated TB‐related miRNAs, and thus might play a role in the pathogenesis of active TB and might represent novel biomarkers for this disease. However, available datasets are limited and these circRNAs signatures should be further confirmed in future studies.

## CONFLICTS OF INTEREST

The authors confirm that there are no conflict of interests.
