# Supplementary material for: Dysregulated circRNAs in plasma from active tuberculosis patients
Source: J Cell Mol Med. 2018 Jun 30;22(9):4076–84. doi: 10.1111/jcmm.13684 (PMC6111852; doi:10.1111/jcmm.13684)
Supplement: Supplementary file 1 [file JCMM-22-4076-s001.docx]

**Supporting information**

**S1 Table** Clinical characters and laboratory measures of the par­ticipants

| Characteristics | BC group (n=32) | CC group (n=29) |
| --- | --- | --- |
| Age (years) | 36.1±13.4 (20-53) | 35.2±12.9 (21-50) |
| Male/female | 17/15 | 15/14 |
| TST test | NA | — |
| IGRA | NA | — |
| HBV | — | — |
| HCV | — | — |
| HIV | — | — |
| Diabetes | — | — |
| History of cancer | — | — |

All newly diagnosed patients with active TB had following clinical symptoms, including 86.1% cough, 75.4% fever, 73.8% night sweats and 68.1% weight loss. All healthy controls and active TB patients were non-smokers. There was no significant difference in age (*P* >0.05) or gender (*P* >0.05) between the different groups. TST, tuberculin skin test; IGRA, Interferon-Gamma release assays; —, negative; BC group: active TB patients group; CC group: healthy control group; NA: not applicable.

**S2 Table** Deregulated circRNAs in active TB group *versu*s controls

| circRNAs | Gene symbol | circRNA type | *P-*value | Fold change | chrom |
| --- | --- | --- | --- | --- | --- |
| hsa_circRNA_104588 | RAB11FIP1 | exonic | 0.004 | 2.993 | chr8 |
| \| hsa_circRNA_065793 \| \| --- \| \| hsa_circRNA_100498 \| \| hsa_circRNA_029301 \| \| hsa_circRNA_404022 \| \| hsa_circRNA_102296 \| \| hsa_circRNA_000222 \| \| hsa_circRNA_085129 \| \| hsa_circRNA_051239 \| \| hsa_circRNA_406174 \| \| hsa_circRNA_048148 \| \| hsa_circRNA_101062 \| \| hsa_circRNA_029965 \| \| hsa_circRNA_104878 \| \| hsa_circRNA_102116 \| \| hsa_circRNA_100823 \| | \| RBM5 \| \| --- \| \| NID1 \| \| NCOR2 \| \| AGPAT6 \| \| ANKRD12 \| \| STAM \| \| ANKRD46 \| \| ATP5SL \| \| PITPNB \| \| CNN2 \| \| CSRNP2 \| \| PDS5B \| \| PTBP3 \| \| ZNF652 \| \| LPXN \| | \| exonic \| \| --- \| \| exonic \| \| exonic \| \| exonic \| \| exonic \| \| exonic \| \| exonic \| \| exonic \| \| sense overlapping \| \| exonic \| \| exonic \| \| exonic \| \| exonic \| \| exonic \| \| exonic \| | \| 0.004 \| \| --- \| \| 0.045 \| \| <0.001 \| \| 0.004 \| \| 0.022 \| \| 0.016 \| \| 0.034 \| \| 0.007 \| \| 0.002 \| \| 0.008 \| \| 0.031 \| \| 0.004 \| \| 0.018 \| \| 0.008 \| \| 0.005 \| | \| 3.175 \| \| --- \| \| 2.535 \| \| 2.123 \| \| 3.985 \| \| 2.268 \| \| 2.002 \| \| 2.104 \| \| 5.358 \| \| 2.934 \| \| 2.990 \| \| 2.046 \| \| 5.174 \| \| 2.099 \| \| 3.809 \| \| 2.286 \| | \| chr3 \| \| --- \| \| chr1 \| \| chr12 \| \| chr8 \| \| chr18 \| \| chr10 \| \| chr8 \| \| chr19 \| \| chr22 \| \| chr19 \| \| chr12 \| \| chr13 \| \| chr9 \| \| chr17 \| \| chr11 \| |
| hsa_circRNA_406841 | MIR5695 | antisense | 0.026 | 2.045 | chr6 |

After normalization of the raw data, we identified 17 circRNAs that were differentially expressed between the two groups by screening for fold-changes greater than 2 and *P* < 0.05. Compared with controls, 16 circRNAs were increased and only one circRNA (hsa_circRNA_406841) was decreased in active TB group.

**S3 Table** KEGG enrichment analysis for hsa_circRNA_103571

| Pathway name | Gene count | *P*-value |
| --- | --- | --- |
| [Ras signaling pathway](https://david.ncifcrf.gov/kegg.jsp?path=hsa04310$Wnt%20signaling%20pathway&termId=550028774&source=kegg) | 47 | 2.9E-4 |
| [Ubiquitin mediated proteolysis](https://david.ncifcrf.gov/kegg.jsp?path=hsa04120$Ubiquitin%20mediated%20proteolysis&termId=550028758&source=kegg) | 32 | 4.3E-4 |
| [Regulation of actin cytoskeleton](https://david.ncifcrf.gov/kegg.jsp?path=hsa04810$Regulation%20of%20actin%20cytoskeleton&termId=550028824&source=kegg) | 41 | 2.9E-3 |
| [Wnt signaling pathway](https://david.ncifcrf.gov/kegg.jsp?path=hsa04310$Wnt%20signaling%20pathway&termId=550028774&source=kegg) | 29 | 4.5E-3 |
| [T cell receptor signaling pathway](https://david.ncifcrf.gov/kegg.jsp?path=hsa04660$T%20cell%20receptor%20signaling%20pathway&termId=550028801&source=kegg) | 22 | 1.2E-2 |
| [Rap1 signaling pathway](https://david.ncifcrf.gov/kegg.jsp?path=hsa04015$Rap1%20signaling%20pathway&termId=550028743&source=kegg) | 37 | 2.2E-2 |
| [PI3K-Akt signaling pathway](https://david.ncifcrf.gov/kegg.jsp?path=hsa04151$PI3K-Akt%20signaling%20pathway&termId=550028768&source=kegg) | 56 | 2.2E-2 |
| [MAPK signaling pathway](https://david.ncifcrf.gov/kegg.jsp?path=hsa04010$MAPK%20signaling%20pathway&termId=550028740&source=kegg) | 43 | 2.6E-2 |
| [HIF-1 signaling pathway](https://david.ncifcrf.gov/kegg.jsp?path=hsa04066$HIF-1%20signaling%20pathway&termId=550028750&source=kegg) | 20 | 2.7E-2 |
| [Insulin signaling pathway](https://david.ncifcrf.gov/kegg.jsp?path=hsa04910$Insulin%20signaling%20pathway&termId=550028825&source=kegg) | 26 | 2.8E-2 |
| [B cell receptor signaling pathway](https://david.ncifcrf.gov/kegg.jsp?path=hsa04662$B%20cell%20receptor%20signaling%20pathway&termId=550028802&source=kegg) | 15 | 3.8E-2 |
